# Use of assessment instruments in forensic evaluations of criminal responsibility in Norway

**DOI:** 10.1186/s12888-022-03831-4

**Published:** 2022-04-01

**Authors:** Pia Jorde Løvgren, Petter Laake, Solveig Klæbo Reitan, Kjersti Narud

**Affiliations:** 1 Faculty of Medicine, University of Oslo, Oslo, Norway; 2 The Regional Centre for Research and Education in Forensic Psychiatry for the South-Eastern Norway Regional Health Authority, Oslo University Hospital, Ullevål Hospital, P. O. Box 4959 Nydalen, 0424 Oslo, Norway; 3 Oslo Centre for Biostatistics and Epidemiology, Department of Biostatistics, University of Oslo, Oslo, Norway; 4 NTNU Faculty of Medicine and Health Science, Department of Mental Health, Trondheim, Norway

**Keywords:** Criminal responsibility, Forensic psychiatry, Insanity, Assessment tool, Psychotic disorders

## Abstract

**Objectives:**

Assessment instruments are often used to enhance quality and objectivity in therapeutic and legal settings. We aimed to explore the use of instruments in Norwegian reports of forensic evaluations of criminal responsibility; specifically, whether this use was associated with diagnostic and forensic conclusions.

**Methods:**

Our study has an exploratory cross-sectional design. We examined 500 reports filed with the Norwegian Board of Forensic Medicine in 2009–2018 regarding defendants indicted for the most serious violent crimes. The first author coded data from all reports according to a registration form developed for this study. Two co-authors then coded a random sample of 50 reports, and inter-rater reliability measures were calculated. The first author coded 41 reports for calculation of intra-rater reliability. Descriptive statistics are presented for the use of assessment instruments, and a generalized linear mixed model (GLMM) was used to estimate associations between the use of instruments and diagnostic and forensic conclusions.

**Results:**

Instruments were used in 50.0% of reports. The Wechler’s Adult Intelligence Scale (WAIS), Historical Clinical Risk-20 (HCR-20), and the Structured Clinical Interview for DSM disorders (SCID I), were used in 15.8, 13.8, and 9.0% of reports, respectively. The use of instruments increased from 36% in 2009 to 58% in 2015; then decreased to 49% in 2018. Teams of two experts wrote 98.0% of reports, and 43.4% of these teams comprised two psychiatrists. In 20.0% of reports, the diagnostic conclusion was schizophrenia, and in 8.8% it was other psychotic disorders. A conclusion of criminal irresponsibility was given in 25.8% of reports. Instruments were more often used in reports written by teams that comprised both a psychiatrist and a psychologist, compared to reports by two psychiatrists. The use of instruments was strongly associated with both diagnostic and forensic conclusions.

**Conclusion:**

Instruments were used in 50% of reports on forensic evaluations of criminal responsibility in Norway, and their use increased during the study period. Use of instruments was associated with diagnostic and forensic conclusions.

## Introduction

In most countries, when there is a question of criminal responsibility (CR), psychiatrists or psychologists provide evidence to the court regarding a defendant’s mental state at the time of the alleged offense. Such evaluations are perhaps the most challenging for mental health professionals [1, 2]. In most jurisdictions, an opinion of criminal irresponsibility can only be reached if a severe mental disorder is present, but not all disorders qualify [3–5]. Studies have shown that psychotic disorders are most often connected to CR [1, 4–11], but even when there is proof of a severe mental disorder, most penal codes require that a connection be made between the disorder and the defendant’s actions at the time of the offense [7, 12]. Thus, a thorough investigation of the defendant’s mental state at time of alleged offense is crucial.

The penal code from 2002 in Norway referred to three legal constructs consistent with criminal irresponsibility, and three constructs consistent with reduced responsibility, which could lead to mitigating circumstances and a lower penalty. Each of these groups of constructs has three prongs: impaired reality testing, intellectual impairment, and reduced mental awareness (Table 1). In contrast to the clinical construct “psychotic”, the legal construct is, for all practical purposes, equivalent to the construct “legally insane”, and we use the latter term in this paper to ensure its differentiation from the clinical term. To be evaluated as legally insane, a defendant must have psychotic symptoms of a certain severity and some functional impairment at the time of the crime, but no connection needed to be established with the offense committed as Norway held the biological / medical principle^1^ [13–15]. The legal construct of severe mental retardation involved intellectual impairment, which could be caused by clinical mental retardation or by other conditions, such as acquired brain damage. The International Classification of Diseases, Revision 10 [16] defines mental retardation as an IQ-level below 70. However, Norwegian legislation dictated that an IQ-level below 75 with functional impairment was sufficient to fulfil the legal construct “less severe mental retardation”. Similarly, severe clinical mental retardation in the ICD-10 is defined as an IQ-level below 50, but Norwegian legislation defined the legal construct “severe mental retardation” as an IQ-level below 55. The legal construct “disturbed consciousness” was comparable to the construct “automatism”, which is known in international forensic literature.

**Table 1:** Legal constructs in the Norwegian penal code on criminal responsibility^a^

		Impairment		
		Impaired reality testing	Intellectual impairment	Reduced mental awareness
Degree of responsibility	Criminal irresponsibility	Psychotic (legally insane)	Severe mental retardation	Strong disturbance of consciousness^b^
	Reduced responsibility	Severe mental illness, but not psychotic	Less severe mental retardation	Less strong disturbance of consciousness

^a^The penal code had three prongs for criminal irresponsibility, and three prongs for “reduced responsibility” (or more correctly stated: mental disorder that could lead to a lesser sentence)

^b^Almost the same as “automatism”

In Norway, the police and the courts have the burden of proof when it comes to CR. When there are indications that a defendant had a mental condition at the time of the offense that might affect their CR, the court appoints a team of experts to perform an evaluation. Mainly, either two psychiatrists or one psychiatrist and one psychologist are appointed. The experts perform independent evaluations and author a joint report which is sent to the court. A copy of the report is sent to the Norwegian Board of Forensic Medicine (NBFM) for quality assessment. The NBFM sends the results of the assessment to the court and to the experts. If the NBFM finds that the report does not meet the quality standards, the experts could be asked to write an additional report that elaborates on any shortcomings. If the shortcomings are less serious, the NBFM quality assessment is presented as additional evidence in court. If the report meets the quality standard, the NBFM responds: “There are no shortcomings in this report”.

Traditionally, psychiatrists have performed evaluations of CR. Psychology emerged as a discipline in the 19th and twentieth century based to a large degree on different forms of testing of human features and abilities. Also when entering the field of forensic evaluations after the second world war [17, 18], psychologists have implemented the use of different test procedures to greater extent than psychiatrists [19].

The retrospective nature of CR evaluations means the approach to diagnosis and evaluation must be different from that used in other forensic or clinical evaluations. In clinical settings, the patient usually seeks treatment, and the experts, together with the patient, explore the patient’s current mental functioning [20]. The core of a CR evaluation is the defendant’s mental state at the time of the offense. Thus, forensic experts have to act more as investigators than clinicians, and they need to collect information from more sources than just the defendants themselves [4, 21]. Many recommend that forensic experts performing CR evaluations should include the following sources: (a) criminal records, records from the time of the offense, (b) interviews with the defendant, (c) use of assessment instruments or other tests, (d) medical records, (e) information from friends, family, and other collateral sources [20, 22].

Many assessment instruments have been developed for use in therapeutic or research settings; few are intended for use in CR evaluations [23], and unlike third-party information, there is less consensus on whether to use tests, and if so, which should be used [24]. Clinical assessment including the use of assessment instruments or tests mainly collects information to confirm or refute current psychological issues and psychiatric diagnoses. This might be relevant for CR evaluations if the results are compared to information regarding the defendant’s behavior, thoughts, and emotions from the time around the alleged offense [20, 21, 23, 25].

In order to achieve a higher quality of forensic assessment of responsibility at the time of an alleged offense, more must be learned about which instruments experts use in their work and what other information they rely on when forming their opinion on the questions asked by the courts [26]. There are two different ways to study this: experts can be asked about their use of and views on the instruments in a survey, or real-world reports or files regarding reports can be studied and coded [27].

We aimed to explore the use of instruments in Norwegian reports of forensic evaluations of criminal responsibility; specifically, whether some instruments are more often used with certain diagnostic or forensic conclusions. We hypothesized that experts used instruments to different degrees depending on their profession, and that usage would increase over the years. We further hypothesized that we would find an association between the use of instruments and diagnostic and forensic conclusions.

## Material and methods

### Study design and material

This is an exploratory, cross-sectional study of registry data. A sample of 500 anonymized reports of forensic mental health evaluations of CR (100 each from 2009, 2011, 2013, 2015, and 2018) was provided from the NBFM secretary. The inclusion criterion was indictment due to the most serious violent crime, thus reports regarding cases of murder and attempted murder were included first. Thereafter, reports regarding less serious indictments, like violence and violent threats were included, and finally sexual crimes were included if necessary to reach the threshold of 100 reports each year. We chose this criterion based on the serious consequences for the offenders and for society.

Between May 2019 and May 2021, the main author (PJL) read all 500 reports and coded the data therein, according to a registration form designed specifically for this study. As the reports from the five study years were not all available at the same time, the first author assessed the reports in the following sequence: 2009, 2015, 2011, 2013, and 2018. Two to 6 months after the first assessment, the first author re-assessed 5–10 reports from each year, to ensure the recordings did not vary over time.

All procedures were performed in accordance with relevant guidelines in the declaration of Helsinki [28].

### Interrater reliability measures

Two of the co-authors (SKR and KN) read and coded 50 randomly selected reports initially coded by the first author, and inter-rater reliability measures were calculated. With an assumed kappa of 0.7 and a 95% confidence interval of 0.5 to 0.9, a sample size of 42 would give sufficient precision. Inter-rater reliability between the three assessors was calculated with Gwet’s AC_1_ [29]. Although other kappa measures have been suggested, they are less desirable when there is high degree of agreement in one category [30]. Gwet’s AC_1_ does not have this undesirable property, thus it was preferred in our case. The first author recoded 41 randomly selected reports, and Cohen’s kappa was calculated.

### Use of assessment instruments

Information on the use of any instrument and of selected, specific instruments (the Wechsler Adult Intelligence Scale (WAIS), diagnostic instruments, the Positive and Negative Syndrome Scale (PANSS), and risk assessment instruments was taken from the reports and categorized as “used” or “not used”.

#### Wechsler Adult Intelligence Scale

The WAIS [31] was included as it is the most common instrument used to assess cognitive functioning in clinical settings in Norway [32].

#### Structured interviews

Structured interviews included the Mini International Interview (MINI) [33], the Structured Clinical Interview for DSM disorders (SCID I) [34], and the Structured Clinical Interview for DSM disorders, personality assessment (SCID II) [35]. These were chosen as they are general diagnostic interview instruments often used in clinical settings in Norway [32]. If any of these instruments were used, the variable diagnostic instruments were recorded as “used”; if none were used, the variable was categorized as “not used”.

#### Positive And Negative Syndrome Scale

The PANSS [36] was included because it is the most common instrument used for assessing severity of symptoms in schizophrenia and other psychotic disorders, both in Norway and most of Europe [32].

#### Risk assessment instruments

Risk assessment instruments included the Historical Clinical Risk-20 (HCR-20) [37] and Sexual Violence Risk-20 [38]. We chose these as they are the most common risk assessment instruments used in clinical settings in Norway. They are also recommended in forensic settings and often specified in the mandate given by the court. If the experts used either of these instruments, the variable was coded as “used”; if they used neither, the variable was categorized as “not used”.

### Characteristics of experts

Information on the gender and the profession of the experts, together with the allocation of the reports to the experts, was coded.

### Characteristics of reports

We recorded the following report variables: The related indictment, experts’ agreement on the ultimate forensic conclusions, whether the experts conducted clinical interviews with the defendant, whether the defendant cooperated with the experts in the evaluation process, and what type of third-party information the experts collected and referred to in the report.

### Diagnostic conclusions

The main diagnostic conclusions were categorized according to the ICD-10 code cited in the reports: no diagnoses, schizophrenia (F20.0-F20.9), other psychotic disorders (F21-F29), affective disorders (F30–39), substance use disorders (F10-F19), personality disorders (F60.0–60.9, F61, and F62), mental retardation (F70–79), and other diagnoses (all other ICD-10 diagnoses). When the conclusions contained several diagnoses, only the main one was used in the analyses.

### Forensic conclusions

Reports with negative conclusions on all legal constructs were categorized as having negative conclusions. Reports with positive conclusions regarding the legal construct psychotic were categorized as legally insane; those with positive conclusions on the constructs severe and less severe mental retardation were categorized as mental retardation. Reports with positive conclusions on the constructs strong and less strong disturbance of consciousness were categorized as disturbed consciousness, and reports with positive conclusions on the construct severe mental disorder but not psychotic were categorized as severe mental disorder.

### Statistical analysis

Descriptive statistics are presented as counts and percentages. Because courts almost always appoint teams of experts to create reports, often the same teams are assigned to write more than one report. However, since the experts contribute to more than one report, there is will be dependence for the assessments in the reports within each team. Associations between the use of assessment instruments and profession are estimated in five 2 × 2 tables, with calculation of odds ratios (ORs). Similarly, associations between the use of assessment instruments and diagnostic and forensic conclusions are presented in five 2 × 8 and five 2 × 7 contingency tables, respectively, also with calculations of ORs. Association between the use of assessment instruments and time, controlling for profession, was estimated by logistic regression. Due to dependence between the reports within teams, odds ratios, 95% confidence intervals, *p*-values were estimated in a generalized linear mixed model (GLMM), with logit link. The data were analyzed with IBM SPSS Statistics tools version 25, and the GLMM in STATA16. A significance level of 5% was used.

### Ethics

The Regional Ethics Committee for Medical Research Ethics in South-Eastern Norway Regional Health Authority (REC) evaluated the study and decided that it was outside the scope of the Health Research Act (2014/539). The Ministry of Justice, by their Council of Confidentiality and Research, and the Office of the Attorney General approved the study. The NBFM encouraged the project and granted access to their archives after anonymization of the reports of forensic evaluations of CR. Permission to inspect the reports was given in accordance with the Public Administration Act § 13 d and Section 63 of the Courts Act. The Data Protection Officer at Oslo University Hospital gave its recommendation to the study (case number 2015/2498). No personally identifiable or demographic data on the defendants were registered. We gave each report and registration form a corresponding ID number, and stored the anonymized data on Oslo University Hospital’s research server.

## Results

A value of Gwet’s AC_1_ between 0.61 and 0.80 is considered to be substantial agreement, and above 0.81 is almost perfect agreement. Interrater reliability for all the assessment instruments and for the forensic and diagnostic conclusions had a Gwets’s AC_1_ value above 0.90 (Table 2). The coding and recoding made by the first author gave Cohen’s kappa values between 0.90 and 1.00 (not shown in table).

**Table 2:** Gwet’s AC_1_ for the study variables

Variables										
	Assessment instruments								Other characteristics	
	At least one instrument	WAIS	MINI	SCID I	SCID II	PANSS	HCR-20	SVR-20	Diagnostic conclusion	Forensic conclusion
Gwet AC_1_	0.95	0.98	0.97	0.98	0.95	1.00	0.91	0.99	0.92	0.94
95% CI	0.87–1.00	0.94–1.00	0.93–1.00	0.95–1.00	0.90–1.00	1.00–1.00	0.83–0.99	0.96–1.00	0.86–0.99	0.87–0.99

**Fig. 1:**
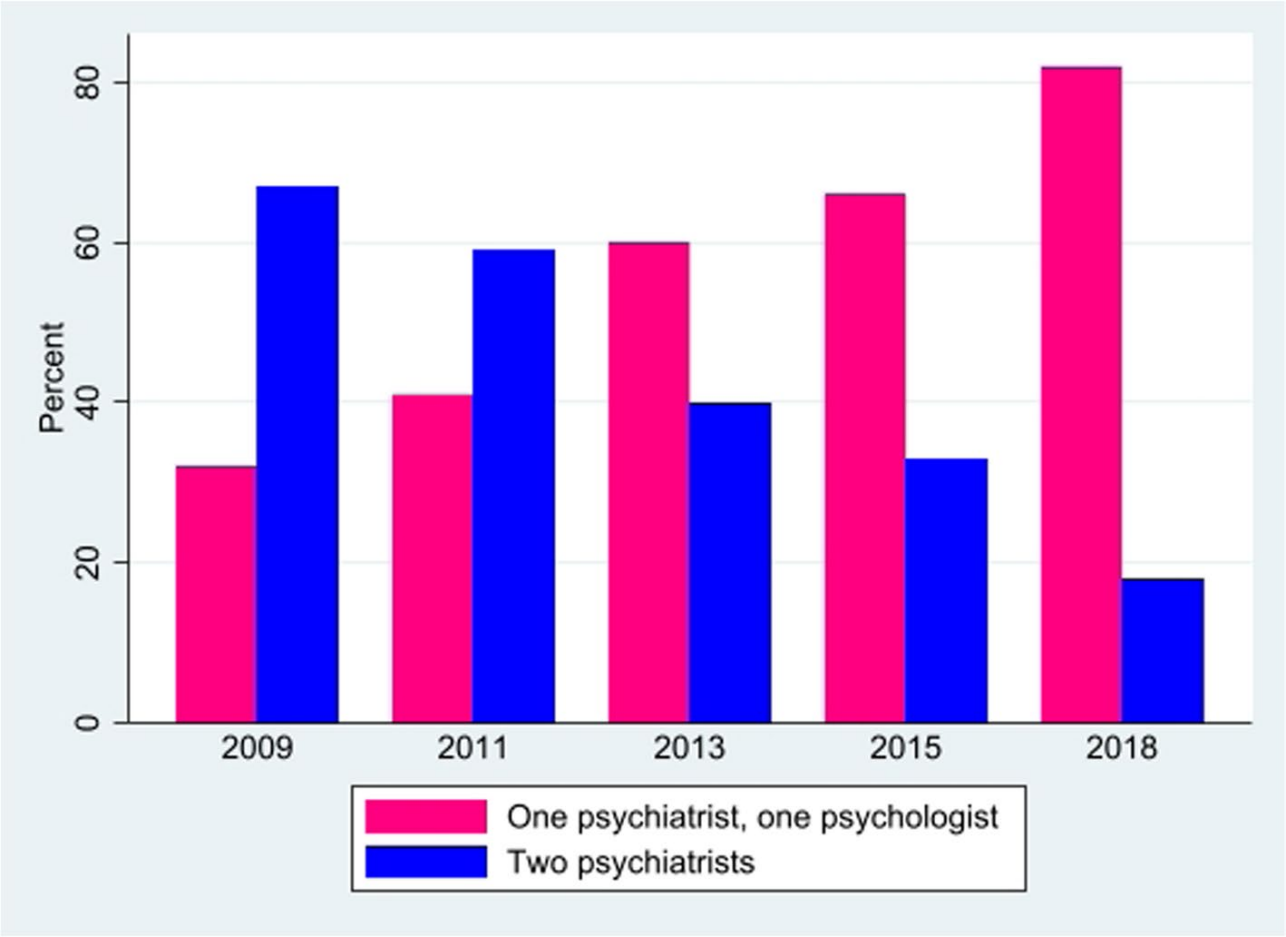
Composition of teams of experts by profession and by year, in percent, *n* = 488^1^. ^1^Selected all reports with two experts, one report written by two psychologists was excluded

Teams of two experts wrote 98.0% of the reports. The total number of non-unique experts writing the 500 reports in the sample was 1005: 718 psychiatrists, 283 psychologists, and four other specialists (one in internal medicine, two neurologists, and one toxicologist). The teams consisted of 138 unique experts. Teams consisting of psychiatrists and psychologists wrote 56.2% of all reports, and the number written by teams consisting only of psychiatrists decreased from more than 60% in 2009 to lower than 20% in 2018 (Fig. 1). Among all reports, 64.8% were written by teams consisting of male experts only (Table 3). Five individual experts were most represented, contributing to 50, 44, 41, 39, and 38 reports, respectively, while 34 experts contributed to one report each (not shown in table).

**Table 3:** Characteristics of experts, cases, and reports

Variables	*n*	(%)
Number of reports	500	
Number of experts^a^	138	(100)
Male	96	(69.6)
Female	42	(30.4)
Total number of experts^b^	1005	(100)
Gender		
Male	810	(80.6)
Female	195	(19.4)
Profession		
Psychiatrists	718	(71.4)
Psychologists	283	(28.2)
Specialist internal medicine	1	–
Specialist neurology	2	–
Specialist toxicology	1	–
Number of unique teams of experts	243	
Number of experts per report		
1	3	(0.6)
2	490	(98.0)
3	6	(1.2)
4	1	(0.2)
Profession per report		
Only psychiatrists	217	(43.4)
Psychologists and psychiatrists	281	(56.2)
Only psychologists	2	(0.4)
Gender per report		
Only male experts	324	(64.8)
Both male and female experts	157	(31.4)
Only female experts	19	(3.8)
Main indictment^c^		
Murder	79	(15.8)
Attempted murder	72	(14.4)
Violence or violent threat	269	(54.8)
Sexual crime	63	(12.8)
Other crimes	7	(1.4)
Agreement between experts	496	(99.2)
Clinical interview with defendant	480	(96.0)
Defendant cooperated with experts	454	(90.8)
Third party information collected		
Police records from current crime	500	(100)
Health records hospitals	361	(72.2)
Health records GP^d^	155	(31.0)
Interview with family and friends^e^	98	(19.6)
Prison records	68	(13.6)
School records	10	(2.0)

^a^Number of unique, individual experts in the sample

^b^Total number of experts in the whole sample

^c^This refers to the most severe indictment if there are several violations of different penal codes

^d^General practitioner

^e^Including spouses and former spouses

Of the 238 unique teams, the five teams that authored the most reports wrote 29, 15, 14, 14, and 12 reports, respectively, of the total sample (numbers not shown in tables). Experts agreed in 99.2% of the reports, and all reports collected at least one kind of third-party information. Almost all reports, 96.0%, included a clinical interview with the defendant (Table 3).

In 20.0% of the reports, the main diagnostic conclusion was schizophrenia; in 8.8%, it was other psychotic disorders; and in 11.8%, the experts concluded there was no diagnosis. In 52.4% of reports, the experts’ determination was that none of the requirements for criminal irresponsibility or reduced responsibility had been met. The most frequent forensic conclusion suggesting criminal irresponsibility was “legally insane”, reached in 23.6% of reports, while 2% was irresponsible due to a severe mental retardation and 0.2% due to a strong disturbance of consciousness (Table 4).

**Table 4:** Diagnostic and forensic conclusions by year

Variables	2009 *n* (%)	2011 *n* (%)	2013 *n* (%)	2015 *n* (%)	2018 *n* (%)	All years *n* (%)
Main diagnosis						
No diagnosis	10 (10.0)	14 (14.0)	10 (10.0)	17 (17.0)	8 (8.0)	59 (11.8)
Schizophrenia (F20.0–20.9)	23 (23.0)	17 (17.0)	15 (15.0)	21 (21.0)	24 (24.0)	100 (20.0)
Other psychotic disorders (F21-F29)	8 (8.0)	9 (9.0)	7 (7.0)	10 (10.0)	10 (10.0)	44 (8.8)
Affective disorders (F30–39)	7 (7.0)	5 (5.0)	14 (14.0)	4 (4.0)	4 (4.0)	34 (6.8)
Substance use disorders (F10-F19)	21 (21.0)	28 (28.0)	23 (23.0)	18 (18.0)	36 (36.0)	126 (25.2)
Personality disorders (F60.0–60.9)	14 (14.0)	14 (14.0)	10 (10.0)	10 (10.0)	6 (6.0)	54 (10.8)
Mental retardation (F70–79)	10 (10.0)	3 (3.0)	6 (6.0)	10 (10.0)	2 (2.0)	31 (6.2)
Others^a^	7 (7.0)	10 (10.0)	15 (15.0)	10 (10.0)	10 (10.0)	52 (0.4)
Total	100	100	100	100	100	500
Forensic conclusions						
Negative conclusion	38 (38.0)	62 (62.0)	63 (63.0)	49 (49.0)	48 (48.0)	260 (52.0)
Legally insane^b^	29 (29.0)	20 (20.0)	18 (18.0)	21 (21.0)	30 (30.0)	118 (23.6)
Mental retardation	11 (11.0)	6 (6.0)	5 (5.0)	10 (10.0)	10 (10.0)	42^c^ (8.4)
Disturbance of consciousness	14 (14.0)	7 (7.0)	11 (11.0)	7 (7.0)	4 (4.0)	43^d^ (8.6)
Severe mental disorder	7 (7.0)	4 (4.0)	1 (1.0)	8 (8.0)	4 (4)	24 (4.8)
No conclusion	1 (1.0)	1 (1.0)	2 (2.0)	5 (5.0)	4 (4)	13 (2.6)
Total	100	100	100	100	100	500

^a^Other categories: Hyperkinetic disorder (F90-), Pervasive developmental disorders (F84-), Organic mental disorders (F0-), Diabetes Mellitus (E10), Parkinson disease (G20), Epilepsy (G40), Cerebral palsy (G80)

^b^“Psychotic” in Norwegian legislation

^c^10 (2.0%) irresponsible (severe mental retardation), 32 (6.4%) reduced responsibility (mild mental retardation)

^d^1 (0.2%) irresponsible (strong disturbance of consciousness),42 (8.4%) reduced responsibility (less strong disturbance of consciousness)

Experts used at least one assessment instrument in 50.0% of the reports, but the proportion varied, starting at 36.0% in 2009, increasing to 58.0% in 2015, and decreasing slightly to 49.0% in 2018. WAIS was the instrument most frequently used (15.8%), followed by HCR-20 (13.8%). Experts used PANSS in 6.3% of the reports (Table 5).

**Table 5:** Use of assessment instruments by year

Variables	2009 *n* = 100	2011 *n* = 100	2013 *n* = 100	2015 *n* = 100	2018 *n* = 100	All years *n* = 500
	*n* (%^a^)	*n* (%)	*n* (%)	*n* (%)	*n* (%)	*n* (%)
At least one instrument	36 (36.0)	50 (50.0)	57 (57.0)	58 (58.0)	49 (49.0)	250 (50.0)
WAIS Wechler Adult Intelligence Scale	14 (14.0)	11 (11.0)	17 (17.0)	21 (21.0)	16 (16.0)	79 (15.8)
MINI Mini International Neuropsychiatric Interview	2 (2.0)	6 (6.0)	11 (11.0)	13 (13.0)	1 (1.0)	33 (6.6)
SCID I Structured Clinical Interview for DSM disorders I	13 (13.0)	13 (13.0)	12 (12.0)	3 (3.0)	4 (4.0)	45 (9.0)
SCID II Structured Clinical Interview for DSM disorders II	2 (2.0)	9 (9.0)	7 (7.0)	4 (4.0)	5 (5.0)	27 (5.4)
PANSS Positive and Negative Syndrome Scale	5 (5.0)	4 (4.0)	6 (6.0)	10 (10.0)	6 (6.0)	31 (6.2)
HCR-20 Historical Clinical Risk Assessment-20	4 (4.0)	20 (20.0)	13 (13.0)	12 (12.0)	20 (20.0)	69 (13.8)
SVR-20 Sexual Violence Risk-20	0	2 (2.0)	1 (1.0)	4 (4.0)	1 (1.0)	8 (1.6)
SCL-90 Symptom CheckList-90	4 (4.0)	6 (6.0)	9 (9.0)	6 (6.0)	1 (1.0)	26 (5.2)
WCST Wisconsin Card Sorting Test	4 (4.0)	2 (2.0)	3 (3.0)	10 (10.0)	6 (6.0)	25 (5.0)
PCL-SV Psychopathy CheckList -Short version	3 (3.0)	10 (10.0)	5 (5.0)	0	2 (2.0)	20 (4.0)
MMPI Minnesota Multiphasic Personality Inventory	0	3 (3.0)	4 (4.0)	7 (7.0)	3 (3.0)	17 (3.4)
PDQ-4 Personality Diagnostic Questionnaire	1 (1.0)	5 (5.0)	4 (4.0)	2 (2.0)	2 (2.0)	14 (2.8)
TOMM Test Of Memory Malingering	1 (1.0)	1 (1.0)	4 (4.0)	4 (4.0)	3 (3.0)	13 (2.6)

Others: RCFT: 2.6%, VINELAND: 2.2%, KEFS: 2.2%, TMT: 2.2%, AUDIT: 2.2%, DUDIT: 2.0%, SRT: 2.0%, GPT: 2.0%, RAVEN 2.0%, MADRS: 1.8%, CVLT: 1.6%, WCST: 1.4%, MCMI: 1.2%, MR-caput/CT-caput: 1.2%, ASRS: 1.2%, CPT: 1.2%, MMS: 1.0%, ASDI: 1.0%, AQ: 1.0%, WMS: 1.0%, Clock-test: 0.8%, YMRS: 0.6%, WURS: 0.6%, V-RISK 10: 0.6%, RAADS-R:0.6%, BRIEF-V: 0.6%, DIS-Q: 0.6%, DES: 0.6%, KAS: 0.6%, FTT: 0.6%, STROOP: 0.6%, Tower of London: 0.6%, WASI: 0.6%, SARA-SV:0.4%, SIDP: 0.4%, GAF: 0.4%, EEG: 0.4%, CIP: 0.4%, BSPS: 0.4%, BAV-Q:0.4%, ADL: 0.4%, ADI-R: 0.4%, WISC: 0.4%,CT: 0.4%

0.2% (1 report each):TSQ, START, SIPS, SIPP, SIMS, SIMP, SCQ, Rorschach, RBANS, QUIP, PSYRATS, PDS, OBS-Dementia, Neurological ex., MDQ, Knox-Cube test, KDV, IES-R, BPRS, Animal naming test, SID-IV, HAD, PAS, CARDS,PCL-S, Malmo-Mast, Antonovsky, Wright, RVSP, BVMT, TPT, FAS, CalCAP, d2, Conners CATA, SVLT, WNV, LUIAS, BVRT, HVLT, COWAT, CFT, PASAT Leiter-R

^a^Percentage of total reports from each year. Some instruments are used in several reports, so the percentage will not sum up to 100

Teams consisting of two psychiatrists used instruments in 40.8% of their reports, compared to 57.4% in teams with one psychologist and one psychiatrist. Teams with two psychiatrists used structured interviews (MINI, SCIDI or SCID II) more often than teams with one psychologist and one psychiatrist but the difference was not significant when analyzed with regard to the individual teams of experts. WAIS was significantly more often used by teams with both professions, with OR of 6.91 (Table 6).

**Table 6:** Use of assessment instruments by profession, n = 488^a^, odds ratios estimated by GLMM

Variables	Any instrument		Structured interviews^b^		PANSS^c^		WAIS^d^		Risk assessment instruments^e^	
	Used	Not used	Used	Not used	Used	Not used	Used	Not used	Used	Not used
*Profession*										
One psychologist, one psychiatrist	159 (57.4)	118 (42.6)	39 (14.1)	238 (85.9)	20 (7.2)	257 (92.8)	66 (23.8)	211 (76.2)	46 (16.6)	231 (83.4)
Two psychiatrists	86 (40.8)	125 (59.2)	51 (24.2)	160 (75.8)	11 (5.2)	200 (94.8)	11 (5.2)^f^	200 (94.8)	26 (12.3)	185 (87.7)
Total	245 (50.1)	243 (49.9)	90 (18.4)	398 (81.6)	31 (6.3)	457 (93.7)	77 (15.7)	411 (84.3)	72 (14.7)	416 (85.3)
OR	2.65		0.46		1.42^g^		6.91		1.42	
95% Confidence interval	1.47–4.77		0.17–1.27		0.63–3.35		2.98–15.97		0.19–3.81	
*p*-value	0.001		0.13		0.37		< 0.001		0.188	

^a^Selected only reports with two experts, 98% of the sample. One report written by two psychologists was excluded

^b^Mini International Neuropsychiatric Interview, Structured Clinical Interview for DSM disorders I or Structured Clinical Interview for DSM disorders II or any combination of these

^c^Positive And Negative Syndrome Scale

^d^Wechler Adult Intelligence Scale

^e^Historical Clincial Risk-20 or Sexual Violence Risk-20 or both

^f^When WAIS was performed in reports written by two psychiatrists, they always collected external evaluation by a psychologist, that was not appointed as expert by the court

^g^Since convergence is not achieved in GLMM, odds ratio is estimated for independence between the reports within teams

To explore whether the observed use of instruments increased significantly over the years studied, or if the increase was mostly associated with the increased number of psychologists co-authoring reports, we made a logistic regression analysis with year and profession as predictors. We restricted the analysis to reports written by teams of two experts (98% of the sample, one report written by psychologists was excluded). We found that the increase was significantly associated both with profession and with year (results not shown in table). Instruments were used significantly more often in the years 2011, 2013 and 2015 compared to 2009.

When calculating associations between use of any instrument and diagnostic and forensic conclusions, we found highly significant associations between both outcomes and the use of any instrument (*p* < 0.001). Likewise, we found highly significant associations between the diagnostic and forensic conclusions and the use of WAIS (p < 0.001). Experts used WAIS most often when their diagnostic conclusion was mental retardation (OR 176, 95% CI = [32.8–944], p < 0.001). Structured interviews were used significantly more often when the diagnostic conclusion was personality disorder (OR 7.54, 95% CI = [1.61–35.2], *p* = 0.010). Compared to negative conclusions, when the forensic conclusion was legal insanity, experts used structured interviews (OR 0.24, 95% CI = [0.08–0.71], p = 0.010) and WAIS (OR 0.22, 95% CI = [0.08–0.66], *p* = 0.006) less often, and PANSS (OR 4.87, CI = [1.35–17.5], *p* = 0.015) more often. When the forensic conclusion was mental retardation, they used WAIS (OR 25.5, 95% CI = [8.67–75.2], *p* < 0.001) significantly more often (Tables 7 and 8).

**Table 7:** Associations between use of assessment instruments and diagnostic conclusions, odds ratios (OR) estimated by GLMM

	F20.0–20.9 Schizophrenia	F21–29 Other psychotic disorders	F30–39 Affective disorders	F10–19 Substance use disorders	F60.0–60.9 Personality disorders	F70–79 Mental retardation	Others^a^	None	
Any instrument									
OR	1	0.98	1.26	1.39	5.01	1.3	2.69	0.88	*N* = 500
95% CI	-	0.37–2.58	0.43–3.65	0.68–2.86	1.95–1.8	3.55–50.2	1.09–6.66	0.37–2.09	*p* < 0.001
*p*-value	-	0.97	0.68	0.37	0.001	< 0.001	0.032	0.77	ICC = 0.40
Structured interviews^b^									
OR	1	0.54	3.84	3.14	7.54	1.94	2.63	0.98	*N* = 500
95% CI	-	0.11–2.74	0.70–21.2	0.93–10.6	1.61–35.2	0.27–14.3	0.60–11.6	0.22–4.38	*p* = 0.10
*p*-value	-	0.46	0.12	0.065	0.010	0.51	0.20	0.98	ICC = 0.71
PANSS^c^									
OR	1	3.42	0.11	0.14	0.48	0.11	0.07	Empty	*N* = 441
95% CI	-	0.68–17.1	0.003–3.28	0.02–0.98	0.07–3.43	0.004–3.15	0.004–1.16	-	*p* = 0.10
*p*-value	-	0.13	0.20	0.047	0.47	0.19	0.063	-	ICC = 0.71
WAIS^d^									
OR	1	2.52	1.01	5.73	4.91	176	12.6	8.94	*N* = 500
95% CI	-	0.44–14.5	0.09–11.1	1.50–21.8	1.05–23.0	32.8–944	2.96–53.3	2.17–36.9	*p* < 0.001
*p*-value	-	0.30	0.99	0.011	0.043	< 0.001	0.001	0.002	ICC = 0.27
Risk assessment^e^									
OR	1	1.15	1.20	1.80	1.34	2.63	2.14	1.84	*N* = 500
95% CI		0.37–3.60	0.35–4.11	0.81–4.02	0.48–3.75	0.90–7.61	0.83–5.54	0.72–4.71	*p* = 0.62
*p*-value		0.81	0.77	0.15	0.58	0.08	0.12	0.21	ICC = 0.00
Total	100	45	34	126	56	31	52	59	500

^a^Others: Hyperkinetic disorder (F90-), Pervasive developmental disorders (F84-), Organic mental disorders (F0-), Diabetes Mellitus (E10), Parkinson disease (G20), Epilepsy (G40), Cerebral palsy (G80)

^b^Mini International Neuropsychiatric Interview or Structured Clinical Interview for DSM disorders I or Structured Clinical Interview for DSM disorders II or any combination of these

^c^Positive And Negative Syndrome Scale

^d^Wechler Adult Intelligence Scale

^e^Historical Clinical Risk-20 or Sexual Violence Risk-20 or both

**Table 8:** Associations between use of instruments and forensic conclusions, odds ratios (OR) estimated by GLMM

	Negative conclusion	Psychotic	Mental retardation	Disturbed consciousness	Severe mental disorder	No conclusion	
Any instrument							
OR	1	0.59	9.9	0.64	0.30	0.18	*N* = 500
95% CI	-	0.32–1.09	3.09–31.4	0.26–1.6	0.09–1.02	0.04–0.87	*p* < 0.001
*p*-value	-	0.09	< 0.001	0.33	0.05	0.03	ICC = 0.42
Structured interviews^a^							
OR	1	0.24	0.76	0.66	0.63	0.10	*N* = 500
95% CI	-	0.08–0.70	0.17–3.37	0.17–2.65	0.11–3.7	0.01–1.58	*p* = 0.13
*p*-value	-	0.009	0.72	0.56	0.61	0.10	ICC = 0.70
PANSS^b^							
OR	1	4.78	0.53	1.40	1.62	1	*N* = 487
95% CI	-	1.32–17.2	0.04–7.15	0.18–10.8	0.18–14.6	Empty	*p* = 0.14
*p*-value	-	0.017	0.63	0.75	0.67		ICC = 0.65
WAIS^c^							
OR	1	0.22	25.3	0.67	0.20	1	*N* = 487
95% CI	-	0.08–0.65	8.60–74.3	0.21–2.14	0.02–.87	Empty	*p* < 0.001
*p*-value	-	0.006	< 0.001	0.50	0.16		ICC = 0.31
Risk assessment^d^							
OR	1	0.95	1.00	1.38	1.58	1.10	*N* = 500
95% CI	-	0.50–1.78	0.40–2.55	0.59–3.20	0.56–4.51	0.23–5.14	*p* = 0.98
*p*-value	-	0.92	0.99	0.46	0.67	0.91	ICC = 0.00
Total	260	118	42	43	24	13	500

^a^Mini International Neuropsychiatric Interview or Structured Clinical Interview for DSM disorders I or Structured Clinical Interview for DSM disorders II or any combination of these

^b^Positive And Negative Syndrome Scale

^c^Wechsler Adult Intelligence Scale

^d^Historical Clinical Risk-20 or Sexual Violence Risk-20 or both

We found a relatively high degree of dependency, measured by the intraclass correlation coefficient (ICC), within the individual teams of experts. The dependency was highest for diagnostic interviews and PANSS (0.6–0.7). For risk assessment, the observations did not depend on the teams writing the report.

## Discussion

The percentage of reports that gave an opinion of criminally irresponsible in our sample was 25.8%, with 23.6% legally insane and 2.2% other causes for criminal irresponsibility. Other studies have shown varying results, with reported ranges of 7 to 36% [1, 6–9, 11, 39–45]. A range of 12 to 17% seems to be most common, thus our results are in the upper range. Among all reports, 28.8% had a diagnostic conclusion of psychotic disorder, of which 20.0% had schizophrenia. Other studies have also shown that psychotic disorders, and schizophrenia in particular are among the most common disorders in CR evaluations [1, 6–8, 10, 39, 45, 46].

Previous studies on CR evaluations have shown great variations in the use of instruments. In a study from Virginia, instruments were used in 2% of reports [7]. Another study from Virginia and one from Florida included competency to stand trial evaluations, and found instruments were used in 16.4 and 16.0% of reports, respectively [40]. In a study from Europe, 61% of reports used instruments [46]. Cochrane et al. found that psychological testing was used in 20% of reports [8], while Warren found that 22% of psychologists and 6% of psychiatrists used testing [9]. Surveys of experts from multiple countries found varying usage, ranging from 68 to 85% [47–49]. Instruments were shown to be used in 23.3% of CR evaluations in Hawaii [42], and in 25.4% of reports written in capital cases [50]. Thus, the proportion of instrument use in our study is in line with that in other studies. There is a large timespan between these studies.

We found a gradual and significant increase in the use of assessments, from 36% in 2009 to 58% in 2015, with a small dip to 49% in 2018.This is in agreement with Neal and Grisso, who observed greater test usage in later years [47]. However, our results contrast with a study by Lawrence et al., in which the use of instruments decreased from 41-51% in 2003–4 to 16–21% in 2017–18 [1], and with those from two large studies from Virginia, in which 20% of reports referred to instruments in 2004 [9], compared to only 2% in 2017 [7]. Thus, some studies, like our research, indicate an increasing use of test instruments over the years, and others indicate the opposite.

Reports written by teams consisting of psychologists and psychiatrists used instruments more often than teams with psychiatrists only, as we hypothesized, and in line previous findings [8, 9, 27, 49, 51]. Fuger et al. on the other hand, found no difference between the professions when using any instrument, but psychologists used cognitive assessment more often, while psychiatrists used forensic assessment more often [42]. When only psychiatrists do CR evaluations, they may consult a psychologist to do some of the testing [46], as was also done in our sample. This practice was more common in the earlier years, in the later years it was more common for the court to appoint a psychologist in addition to a psychiatrist if cognitive assessment was deemed necessary. The increase we observed in test usage over time remained significant after controlling for profession, and thus cannot be explained by the fact that many more psychologists co-authored reports in 2018 than in 2009.

As has been observed in previous studies WAIS [24, 27, 52] and risk assessment with HCR-20 [47] was common in our sample of reports. The use of other instruments, however, has not been widely reported in the literature. SCID was used often in our sample, but we were only able to find one other study that mentioned it as a means to assess present psychopathology in CR evaluations [4]. We also found no studies that mention PANSS, and this instrument is likely not often used in CR evaluations outside of Norway. One of the most striking differences was related to the MMPI and the Rorschach, instruments that were used rarely in our sample, but that are often used in CR evaluations elsewhere, such as Belgium and the USA [24, 25, 27, 46, 47, 52]. This difference can be explained by the fact that the MMPI and the Rorschach are based on a diagnostic system that is not used in Norway, and their validity in forensic settings is questioned. Thus their use in forensic settings is discouraged in Norwegian textbooks and by the NBFM [32]. Assessment of response style and malingering is often recommended in CR evaluations [23], but it is not performed systematically in Norway, except as a part of assessment of intellectual functioning, for which Test of Memory Malingering, TOMM, is often used.

Norwegian legislation differs from that of most other countries in that there is no need to establish a connection between the defendants’s content of thoughts at the time of the offense and the criminal offense committed. This might increase the usefulness of assessment instruments, as the general functional level of the defendant at the time of the offense is of more interest than what he/she thought or felt at the time.

Previous recommendations have advised experts to use instruments only when they are relevant to the clinical issue in question [21, 23, 26]. We found significant associations between use of instruments and the diagnostic and forensic conclusions in reports. PANSS was used significantly more often when the forensic conclusion was legally insane (“psychotic” in the legal sense). As PANSS measures the severity of symptoms in schizophrenia, and legislation in Norway demands a certain severity of psychotic symptoms at the time of the offense, this seems to be both clinically and legally relevant. WAIS was used significantly more often when the diagnostic or forensic conclusion was mental retardation. Thus, it seems that experts regularly use assessments of intellectual functioning to draw conclusions regarding intellectual impairment. SCID was used more often when personality disorders were diagnosed, as is clinically relevant, but probably more related to risk assessment than to the responsibility issue. Thus, it seems that Norwegian experts in our sample generally followed recommendations to choose tests relevant to the clinical issue. A previous study by Lawrence et al. found no associations between forensic opinions and psychological testing [1], and few other studies have looked into associations between conclusions and testing.

The existing literature suggests that the individual preferences of experts are as important to the use of assessment instruments as the diagnostic or legal issue at hand [27, 50], but this seemed only partly true in our sample. In our analyses of the associations between instruments and conclusions, we found a high ICC for diagnostic interviews (MINI and SCID) and for PANSS. Thus, there was high dependency for teams of experts for these associations. We may speculate that experts probably used these instruments more often due to personal preference, rather than the specific issue in question. Risk assessment instruments, on the other hand, showed no dependency in our sample (ICC = 0.00) which means their use was not dependent on the individual teams of experts.

Current recommendations state that forensic experts evaluating CR should base their opinion on information from many sources [20, 25, 51]. Indeed, in many ways one does not collect the same data in a standard clinical situation as in a CR evaluation, because the latter requires historical information, as well as information on the defendant’s background, current mental status, and mental state at the time of the offense from witnesses, criminal files, and other sources [21, 22]. Most reports in our sample used more than one source of information and the use of multiple sources has been reported in many previous studies [42, 46, 47, 50, 52]. Thus, the quality of Norwegian reports of CR seems to be in line with standards elsewhere.

Studies have shown that forensic experts do not always agree in their evaluations of CR [2, 12, 53, 54]. This is even more evident in jurisdictions that request several evaluations by independent experts or expert teams [43], with reported agreement as low as 55.1% [41]. Therefore, the high agreement that we observed between experts in our sample, as high as 99.2%, is surprising. Experts are supposed to complete independent evaluations and author a joint report, but they do have the opportunity to discuss their conclusions. The low levels of agreement in other studies may indicate that experts in Norway discuss cases and reach a common opinion before writing their reports. This practice may make it easier for the court to rule on the question of CR [43], but it raises concern as to whether the evaluation process provides the court with all relevant information [20]. Indeed, the courts should be presented with all information necessary to reach a decision, including the data, observations and inferences experts used to reach their conclusions [4, 27, 41]. The unanimous nature of evaluations begs the question of whether the experts are really disclosing any possible disagreements they had before reaching their diagnostic and forensic conclusions.

There are differing views on the usefulness of instruments when evaluating CR, with many previous studies advocating a limited role for testing. One textbook from 2018 suggests that forensic experts should not rely too heavily on testing due to the lack of evidence that the resultant data can establish a link between the diagnoses and the legal issue, and the fact that testing only provides information about current functioning, not the defendant’s mental state at time of offense [20]. In a recent study, Lawrence et al. concluded that the use of psychological instruments for CR evaluation is not “a standard expectation in the field”, a finding they believed to be supported by the decrease in instrument use in reports [1]. Other authors maintained that psychological testing is helpful to evaluate a clinical construct, but not necessary to reach a forensic opinion [4, 24]. Indeed, few instruments have been specifically developed to assess CR, with some suggesting that it is not “instrumentable” because it involves retrospective inferences about a past mental state [55]. However, supporters of the usefulness of tests when evaluating CR make the case that instruments may be helpful to create and test hypotheses about a defendant’s diagnostic and clinical status at the time of the evaluation, which could be relevant for their mental state at the time of the offense [21]. Others see testing as a source of hypotheses about psychological constructs that might be relevant for the legal standard, while stressing the importance of other sources [23], or recognize their value in understanding of the defendant’s “psychological makeup” [56], with some even agreeing that formal assessment tools should always be used [46]. An international survey of experts reported that it was encouraging that more than 70% of their study sample used tests of any kind as “structured tools improve clinical decision making” [47]. However, this survey included evaluations of forensic issues other than CR evaluations, where tests might have another role.

There is little empiric evidence that mental disorders diagnosed by the use of tests really give a more accurate evaluation of the offender’s mental state at the time of the offense, or if the use of many sources is more relevant for this purpose [21, 25, 42]. The links between test results and conclusions can be vague and inaccurate [12, 27]. There might also be differences between what other mental health professionals regard as a report of high quality, and what the legal system needs or wants from a report, as different kinds of data could be important for the clinical and legal objectives [57].

### Strengths and weaknesses

The relatively high number of reports is considered a strength of this study, as are the high interrater reliability scores. Many field studies include all reports from a certain period, and most have the weakness that some experts write multiple reports in the sample, while some experts write only a few [7, 46]. This weakness is eliminated in surveys, which take information from each expert only once. However, surveys can only include the information experts disclose, which may reflect their own views more than real world practice. Studying forensic reports directly is seen as an improved methodology over surveys [1] We included information on the individual teams of experts and analyzed the associations between the instruments and the diagnostic and forensic conclusions with nesting of reports within teams of experts, which gives additional information. We found a relatively high ICC, which means that the information is not independent within the expert teams.

Our study was designed to explore the contents of reports of forensic evaluation of CR. It was not designed to give information on whether the use of instruments was associated with higher quality reports. Nor was it possible to validate the diagnostic and forensic conclusions. All studies of CR evaluations share this weakness, as the conclusions depend on a retrospective assessment of a possible mental condition that might have been present at the time of the offense. We did not analyze the setting in which the evaluation was done, which may be of interest, as the defendants most affected by a mental disorder were most likely hospitalized during the ongoing observation. This may have created difficulties in administering tests, but also provided an opportunity for the collection of good observational data that supported the diagnostic conclusions. We did not record information on whether experts gave a justification of which instruments they did or did not use, which might have been interesting; nor did we evaluate the instruments used, if they are seen as acceptable in the field, or if they have the expected reliability and validity [25]. The Mental Measurements Yearbook is a central source for evaluations of psychological assessment instruments and is recommended in several papers [23, 26]. There is no similar source for instruments in regular use in Norway. Moreover, many instruments have not been translated into Norwegian, and therefore are not used.

To the best of our knowledge, the Norwegian system of determining CR, with a national board of forensic medicine that reads, evaluates, and provides feedback on all reports, is unique to Norway. It was established in 1900 and includes all forensic disciplines. We believe this system elevates the standardization of reports substantially and thereby probably to their quality. The remarks and comments from the NBFM are always included when the report and its conclusions are presented at trial, which is reassuring for the court.

## Conclusion

Instruments were used in 50% of reports on forensic evaluations of criminal responsibility in Norway, and their use increased during the study period. Use of instruments was associated with diagnostic and forensic conclusions. The differing viewpoints in the current literature suggest that there is still no consensus on the use of structured assessment instruments in forensic evaluations of CR, even if such instruments have been shown to improve evaluations in other fields of forensic and clinical practice. Further studies should conduct a more in-depth exploration of whether the use of structured assessment instruments is associated with higher quality of assessment of the defendant’s mental state at the time of an offense, as well as to what degree legal consumers find reports that used tests more informative. The goal of increasing quality of evaluations of CR is to ensure that the right persons are judged as criminally irresponsible. Which quality factors contribute most to correctly identifying “true” irresponsible offenders remains an open question. We believe it is important to explore these factors even if the retrospective nature of the evaluation could limit the insights learned from these studies.

## Data Availability

The datasets generated and analyzed during the current study are not publicly available due to the Data protection policy at Oslo University Hospital. They are stored in a secured research server and are available from the corresponding author on reasonable request.

## Footnotes

1 The law was changed in 2020, after the reports in our study were written. In the new legislation, the experts should no longer give a conclusion or opinion on CR, but describe the mental state and functioning level of the defendant at the time of the offense. The construct “psychotic” is replaced by the new legal construct “severely deviant state of mind”.
